# Age-related macular degeneration eyes presenting with cuticular drusen and reticular pseudodrusen

**DOI:** 10.1038/s41598-022-09608-9

**Published:** 2022-04-05

**Authors:** Je Moon Yoon, Dong Hoon Shin, Mingui Kong, Don-Il Ham

**Affiliations:** 1 Department of Ophthalmology, Samsung Medical Center, Sungkyunkwan University School of Medicine, 81 Irwon-ro, Gangnam-gu, Seoul, 06351 Korea; 2 Hangil Eye Hospital Retina Center, Incheon, Korea

**Keywords:** Diseases, Eye diseases, Macular degeneration, Retinal diseases, Vision disorders

## Abstract

This study aimed to describe the clinical characteristics of age-related macular degeneration (AMD) eyes with both cuticular drusen (CD) and reticular pseudodrusen (RPD). Clinical records of patients diagnosed with CD or RPD with multimodal imaging was reviewed for patients diagnosed with both CD and RPD. The distribution patterns of CD (macular and diffuse type) and RPD (localized, intermediate, and diffuse type), presence of soft drusen, large drusen (> 200 µm), variant subretinal drusenoid deposits, and macular complications were investigated. Of the 220 eyes of 110 patients diagnosed with CD and 926 eyes of 463 patients diagnosed with RPD, 13 eyes of seven patients met the diagnostic criteria for both CD and RPD. The mean age at initial presentation was 71.4 ± 8.8 years and six patients were female. The mean subfoveal choroidal thickness was 143.8 ± 25.1 µm. The distribution of CD was of the macular type in all eyes. Distribution of RPD was localized in 11 eyes (84.6%) and intermediate in two eyes (15.4%). Soft drusen, large drusen, and variant subretinal drusenoid deposits were present in 13 (100%), 12 (92.3%) and, seven (53.8%) eyes, respectively. Macular neovascularization was observed in two eyes (15.4%). CD and RPD can coexist in eyes with AMD. Multimodal imaging should be used for AMD eyes with features suggestive of CD and RPD, considering the high likelihood of developing late AMD.

## Introduction

Age-related macular degeneration (AMD) is the most common cause of blindness in older individuals in industrialized countries. Late AMD, including geographic atrophy (GA) and macular neovascularization (MNV), is the final presentation of irreversible vision loss^1^.

Drusen are focal deposits of extracellular debris and are a hallmark of AMD^1^. Many studies, using new imaging methods and histological examinations, have provided further information on drusen. It has been recognized that there are diverse phenotypes of extracellular deposits in AMD eyes, including soft drusen, cuticular drusen (CD), and reticular pseudodrusen (RPD)^2,3^. Interestingly, eyes with CD or RPD showed many imaging and clinical features that differed from those of eyes with only soft drusen.

CD are multiple, small, round drusen that appear hyperfluorescent on fluorescein angiography (FA), showing a “stars-in-the-sky” appearance^4^. CD are widely scattered on the fundus with symmetrical distribution patterns in both eyes. The fundus topographic distribution patterns of CD are classified into macular type and diffuse type according to whether the deposits cross the vascular arcades^2^. An earlier age of onset and stronger genetic association have been reported for AMD eyes with CD, as compared to the rest of the AMD population^5^. CD can cause visual loss as a result of vitelliform macular detachment or late AMD, and could be related to membranoproliferative glomerulonephritis type II and other renal diseases^2^.

Subretinal drusenoid deposits (SDD), also known as RPD, are deposits in the subretinal space between photoreceptors and the retinal pigment epithelium, representing morphological features distinct from those of other subtypes of drusen. They appear as bluish-white dots or net-like patterns in color fundus photography (CFP), and subretinal accumulations on optical coherence tomography (OCT)^3,6^. RPD are risk factors for late AMD, particularly GA and type 3 neovascularization^7,8^. A previous study categorized the fundus topographic distribution pattern of RPD into three types (localized, intermediate, and diffuse type) and the diffuse type was a risk factor for the development of late AMD^9,10^. Also, variant SDD showing imaging features similar to those of RPD except hyperautofluorescence on fundus autofluorescence (FAF) imaging was previously reported^11^.

Although there are many reports on CD and RPD, the concurrent development of CD and RPD in AMD eyes was rarely reported, and many facts are still unclear, including incidence, prevalence, clinical features, and visual prognosis^12,13^. An eye manifesting both types may have an additive risk for the development of late AMD, because each of CD and RPD are associated with late AMD. In the present study, we report AMD eyes representing both CD and RPD, and investigate their clinical characteristics, including multimodal imaging features.

## Results

Of the 220 eyes of 110 patients diagnosed with CD and 926 eyes of 463 patients diagnosed with RPD (Supplementary table 1), 13 eyes (5.9% in CD eyes and 1.4% in RPD eyes) of seven patients were diagnosed with both CD and RPD (Table 1). Only one eye was included in one patient (Case 4), because the other eye did not meet the diagnostic criteria for RPD. Two patients had systemic hypertension, two had diabetes mellitus, and one had hypothyroidism. There was no case of membranoproliferative glomerulonephritis, which were previously described to be associated with CD^6^. However, one patient had chronic kidney disease of unknown origin. The mean age was 71.4 ± 8.8 years (range, 57–80 years), and six patients were female (85.7%). The mean spherical equivalent was 0.13 ± 0.43 diopters (range, − 0.75 to + 0.88 diopters), and BCVA at first visit was 0.2 ± 0.4 logMAR (logarithm of the minimum angle of resolution) (range, 0.0–1.3 logMAR). The average subfoveal choroidal thickness (SFCT) was 143.8 ± 25.1 µm (range, 103–181 µm). Eight eyes were pseudophakic, and five eyes were phakic. Two eyes had mild non-proliferative diabetic retinopathy, and two eyes had normal tension glaucoma.

**Table 1:** Characteristics of eyes presenting with cuticular drusen and reticular pseudodrusen.

	Age (years)	Sex	Laterality	BCVA (logMAR)	SFCT (µm)	Distribution of CD	Distribution of RPD	Soft drusen	Large drusen (> 200 µm)	Variant SDD	Macular complications	Ocular comorbidities
Case 1	73	F	OD	0.2	138	Macula	Localized	+	+	−		
			OS	1.0	176	Macula	Localized	+	+	−		
Case 2	75	F	OD	0.0	131	Macula	Localized	+	+	+		
			OS	0.0	138	Macula	Localized	+	+	+		
Case 3	62	F	OD	0.1	110	Macula	Intermediate	+	+	+		
			OS	0.1	103	Macula	Intermediate	+	+	+		
Case 4	57	M	OS	0.0	143	Macula	Localized	+	−	−		
Case 5	80	F	OD	1.3	141	Macula	Localized	+	+	−	MNV type 1	NPDR
			OS	0.1	176	Macula	Localized	+	+	+	MNV type 1	NPDR
Case 6	73	F	OD	0.0	181	Macula	Localized	+	+	+		
			OS	0.0	171	Macula	Localized	+	+	+		
Case 7	80	F	OD	0.0	134	Macula	Localized	+	+	−		NTG
			OS	0.1	128	Macula	Localized	+	+	−		NTG

BCVA, best-corrected visual acuity; CD, cuticular drusen; logMAR, logarithm of the minimum angle of resolution; MNV, macular neovascularization; NPDR, non-proliferative diabetic retinopathy; NTG, normal tension glaucoma; RPD, reticular pseudodrusen; SDD, subretinal drusenoid deposit; SFCT, subfoveal choroidal thickness; M, male; F, female; OD, right eye; OS, left eye.

### Multimodal imaging characteristics

**Figure 1:**
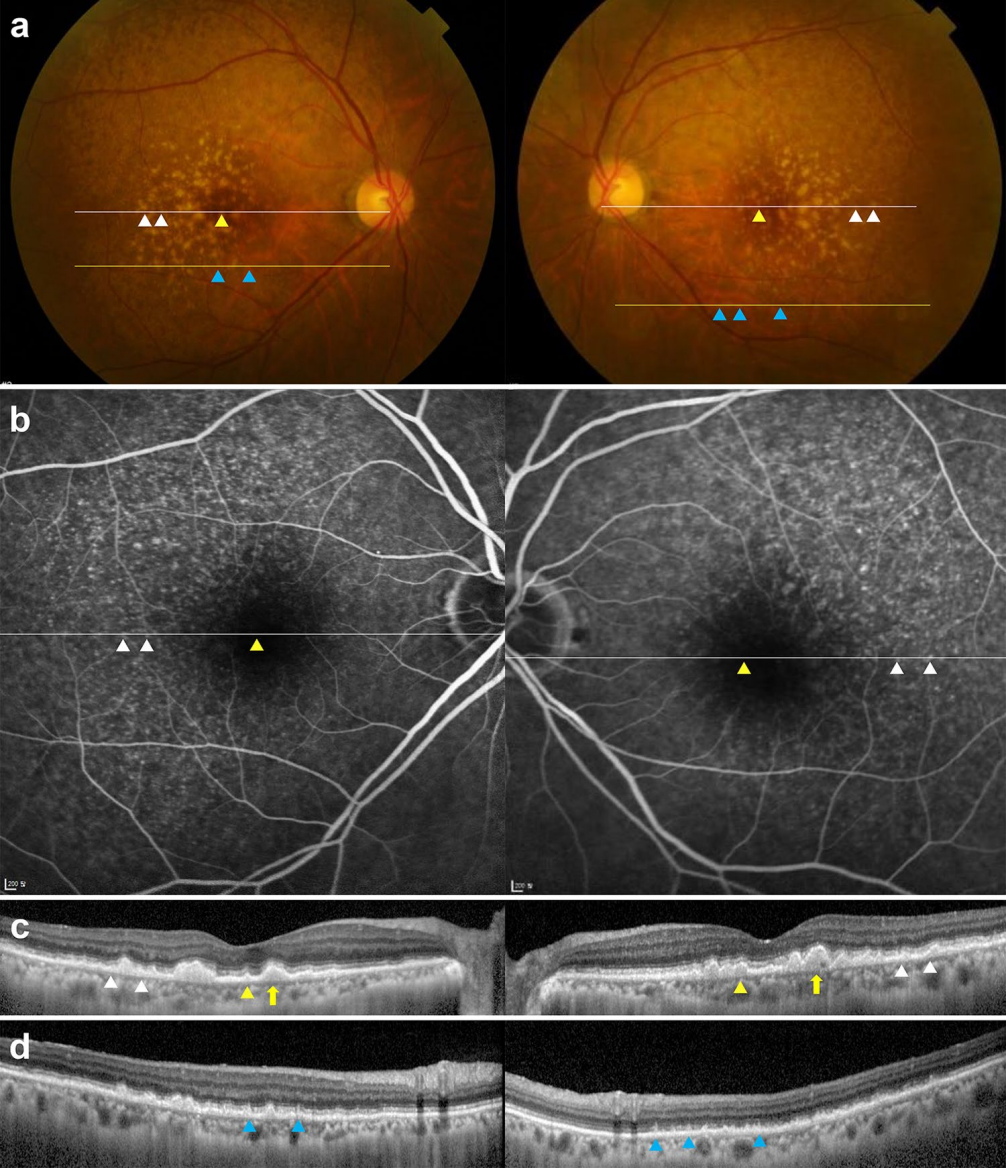
Multimodal Imagings of a 73-year-old woman (Case 1). (**a**) Numerous yellowish and whitish deposits of various sizes are present in color fundus photograph in both eyes. (**b**) Fluorescein angiography (FA) of the venous phase shows multiple hyperfluorescence of “stars-in-the-sky” appearance. (**c**) Optical coherence tomography (OCT) scan (white line in A and B) shows sub-retinal pigment epithelium (RPE) deposits. Some of them shows hyperfluorescence in FA (cuticular drusen, white arrowhead), and others shows hypofluorescence in FA (soft drusen, yellow arrowhead). Large drusen (> 200 μm) is also observed (yellow arrow). (**d**) OCT scan, obtained at the location of the yellow line in A, shows subretinal deposits with a conical appearance, which break through the ellipsoid zone, corresponding to reticular pseudodrusen (blue arrowhead). They have no hyperfluorescence in FA.

**Figure 2:**
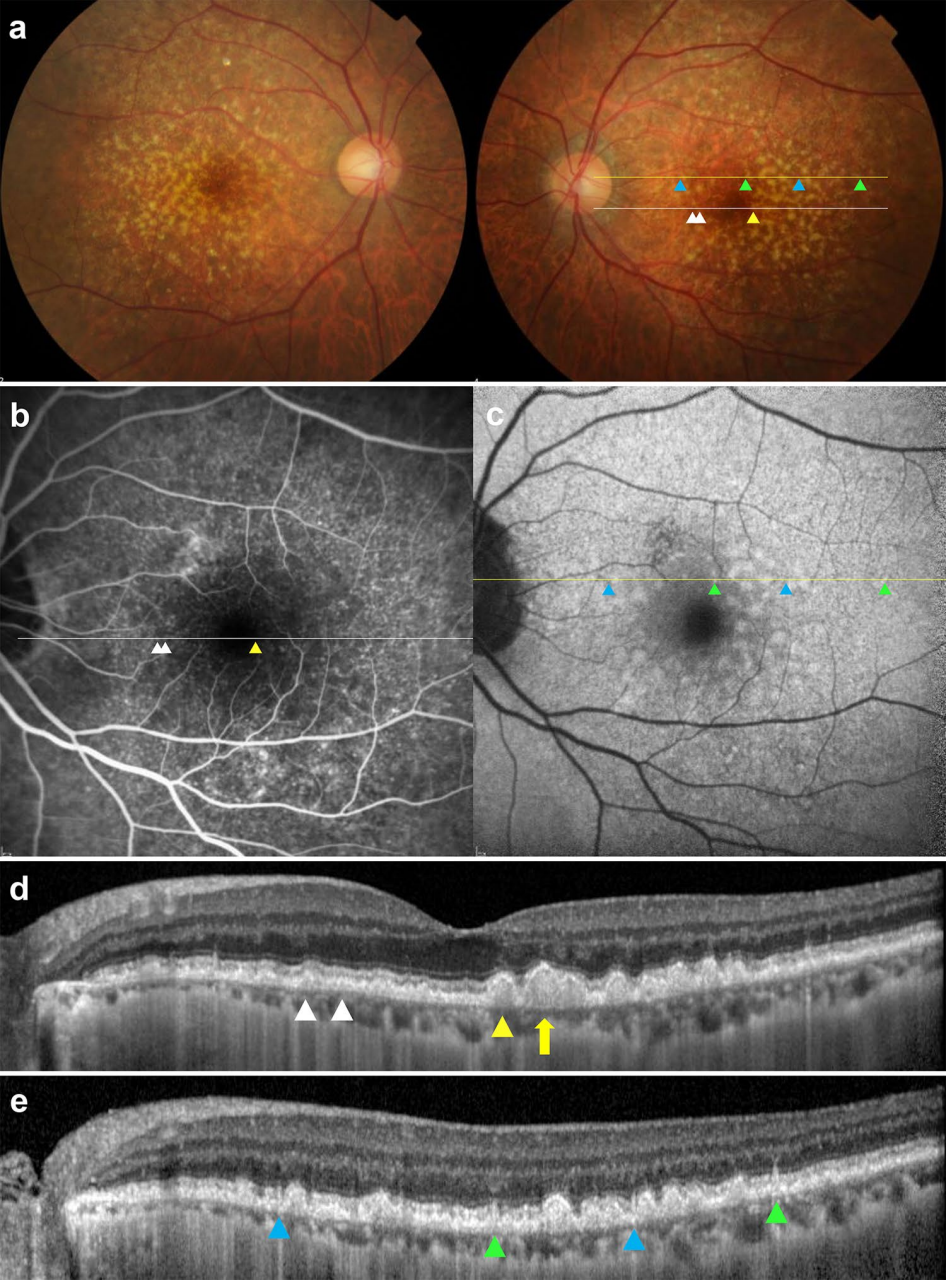
Multimodal Imagings of a 75-year-old woman (Case 2). (**a**) Color fundus photograph shows small to large deposits at the posterior pole in both eyes. (**b**) Fluorescein angiography (FA) of the venous phase shows numerous hyperfluorescent spots, and (**c**) fundus autofluorescence shows numerous hypoautofluorescent centers surrounded by hyperautofluorescent rings. (**d**) Optical coherence tomography (OCT) scan (white line in A and B) shows saw-tooth elevation of retinal pigment epithelium (RPE) at the position of hyperfluorescent spots in FA, corresponding to culticular drusen (white arrowhead). Yellow arrowhead indicates sub-RPE deposit with no hyperfluorescence in FA (soft drusen). Large drusen (> 200 μm) is also observed (yellow arrow). (**e**) OCT scan at the location of the yellow line in A and C shows subretinal deposits, corresponding to reticular pseudodrusen (blue arrowhead) and variant subretinal drusenoid deposits (green arrowhead).

**Figure 3:**
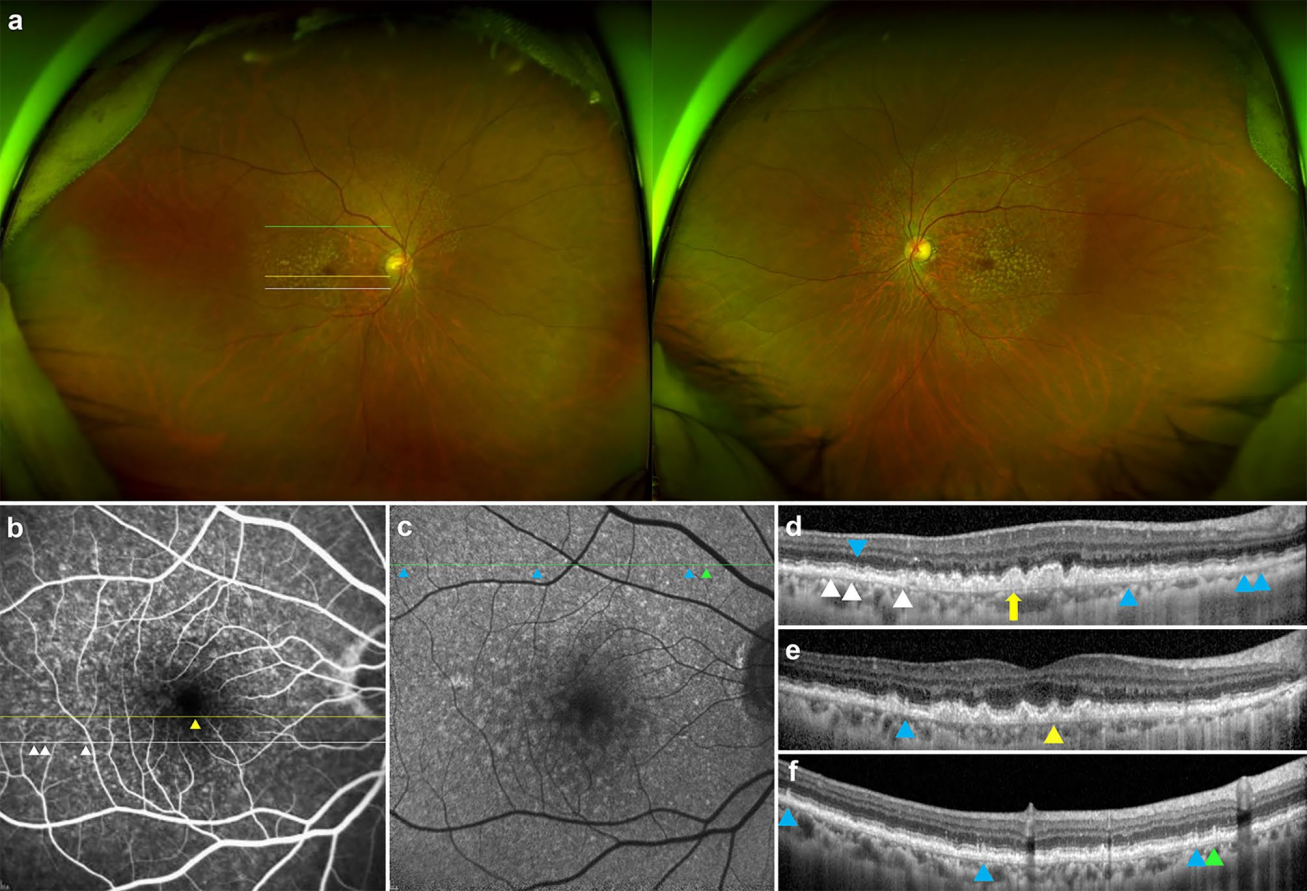
Multimodal imagings of a 62-year-old woman (Case 3). (**a**) Numerous yellowish and whitish deposits of various sizes are present in ultrawide-field photograph, and whitish deposits (reticular pseudodrusen) exist beyond the major arcades in both eyes. (**b**) Fluorescein angiography of the venous phase shows multiple hyperfluorescence of “stars-in-the-sky” appearance within the major vascular arcades. (**c**) Fundus autofluorescence shows numerous hypoaurofluorescent and hyperautofluorescent spots. (**d**) Optical coherence tomography (OCT) scan (white line in A and B) shows saw-tooth elevation of retinal pigment epithelium (RPE) corresponding to cuticular drusen (white arrowhead). Multiple subretinal deposits, corresponding to reticular pseudodrusen (blue arrowhead) and large drusen (yellow arrow, > 200 μm) are also observed. (**e**) OCT scan taken at the position of the yellow line in A and B shows sub-RPE deposit (yellow arrowhead) showing no fluorescence on Image B (soft drusen). A blue arrowhead indicates a reticular pseudodrusen. (**f**) OCT scan, obtained at the location of the green line in A and C, shows subretinal deposits corresponding to reticular pseudodrusen (blue arrowhead) and variant subretinal drusenoid deposits (green arrowhead).

CFP, FAF, near-infrared reflectance (NIR), OCT, ultrawide-field (UWF) photography, FA, and indocyanine green angiography (ICGA) were performed in all eyes. Red-free imaging (RF) was performed in nine (69.2%) eyes. All eyes showed characteristic imaging features of both CD and RPD. In CFP, numerous yellowish and whitish deposits of various sizes were present against a background of pigment disturbance (hypopigmentation and hyperpigmentation) (Figs. 1a, 2a, and 3a). There were larger deposits in the central macular area than in the perimacular area, and small deposits were denser in the superior area than in the inferior area to the macular center. CD appeared as clusters of small, yellow spots, and RPD appeared as slightly bluish-white dots or interlacing networks. However, it was often difficult to distinguish CD from RPD without multimodal imaging where they coexisted, such as at the superior area of the posterior pole (Fig. 1a) on CFP images. In RF imaging, CD appeared as multiple small, whitish dots in all eyes, but they were less numerous than on FA (Fig. S3c). RPD appeared as slightly white lesions but were less bright than CD on RF imaging. In NIR imaging, CD showed heterogeneous reflectivity, whereas RPD showed discrete hyporeflectivity with target configurations (Fig. S2f.). In FAF imaging, CD appeared as punctate hypoautofluorescent lesions with a ring of hyperautofluorescence in 8 of 13 eyes (61.5%) (Fig. 2c and 3c). RPD appeared as hypoautofluorescent lesions in all study eyes, and variant hyperautofluorescent SDD were observed in seven eyes (53.8%) (Fig. 2c and 3c).

**Figure 4:**
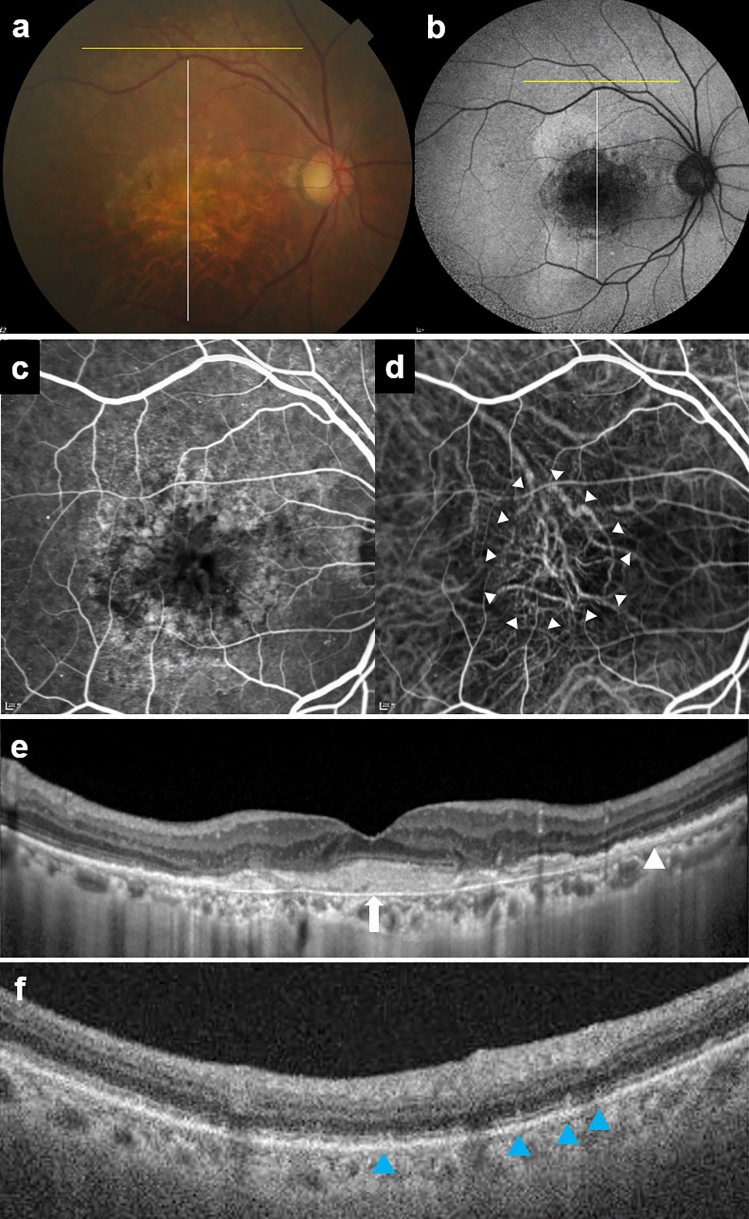
Multimodal imagings of an 80-year-old woman who had macular neovascularization and non-proliferative diabetic retinopathy (Case 5). (**a**) Multiple deposits of various sizes are present at the macula and the area superior to the macula in color fundus photography. Macular hypopigmentation is also observed. (**b**) Fundus autofluorescence imaging shows floodplain hyperautofluorescence. (**c**) Fluorescein angiography of the venous phase shows perifoveal hyperfluorescent areas, microaneurysms, and small hyperfluorescent spots corresponding to cuticular drusen. (**d**) Indocyanine green angiography (ICGA) shows neovascularization (white arrowheads). (**e**) Optical coherence tomography (OCT) scan, obtained at the location of the white line in A and B, shows saw-tooth elevation of retinal pigment epithelium (white arrowhead), subretinal hyperreflective material (white arrow), and pigment epithelial detachment. (**f**) OCT scan, obtained at the position of the yellow line in A and B, shows subretinal deposits corresponding to reticular pseudodrusen (blue arrowhead).

The morphological features of CD seen on OCT B-scans can be broadly categorized into two patterns. Three eyes (23.1%) showed shallow elevations of the RPE–basal laminar band, with druse internal contents being difficult to discern (Fig. 4e). Ten eyes (76.9%) showed drusen with triangular morphology, giving a sawtooth appearance, and hyporeflective internal contents. The morphological features of SDD on OCT B-scans were subretinal deposits with a conical appearance, which occasionally broke through the ellipsoid zone (Fig. 1d).

FA showed numerous tiny hyperfluorescent spots, akin to a “stars-in-the-sky” appearance, for CD not for RPD (Fig. 1b). Discrete hyperfluorescence typically occurred in the early arteriovenous frames and persisted throughout the late frames of the angiogram. CD appeared smaller and less numerous on ICGA than on FA. In ICGA, RPD appeared hypofluorescent in all eyes.

The topographic distribution of CD was of the macular type in all eyes. The topographic distribution of RPD was of the localized, intermediate, and diffuse type in 84.6% (11/13), 15.4% (2/13), and 0% (0/13) of the eyes, respectively.

By analyzing multimodal imaging, soft drusen and large drusen were present in 13 eyes (100%) and 12 eyes (92.3%) of the 7 patients, respectively.

Two eyes (15.4%) had a type 1 MNV and underwent intravitreal injection of anti-vascular endothelial growth factor one year ago (Fig. 4). There were no cases associated with GA, drusenoid PED, or AVL.

Multimodal imaging results of Case 4, 6, and 7 are shown in the Supplementary Figs. S1–S3).

## Discussion

The current study showed that CD and RPD coexist in some eyes with AMD. Although Sakurada et al. published photographs of CD presenting along with RPD, there were no reports on case series of AMD eyes with both CD and RPD in a literature search in PubMed^12^. Since the annual incidence rate of RPD was reported to be only 2.9%, and the incidence rate of CD is expected to be even lower, the probability of the two being together may be very small^14^. Only seven cases were found in 110 CD or 463 RPD patients in this study (5.9% in CD eyes and 1.4% in RPD eyes).

Patients simultaneously presenting both CD and RPD had demographic features similar to those of patients with CD or RPD. The mean age of the seven patients with CD and RPD was 71.4 ± 8.8 years. Compared with previous reports of a Korean population, the mean age of this cohort was older than patients with CD (66.6 ± 9.1 years) and younger than patients with RPD (72.6 ± 9.0 years)^15,16^. This cohort showed a female preponderance (85.7%), similar to Korean patients with CD (82.7%) or with RPD (86.2%)^15,16^.

Although eyes with CD and RPD showed imaging features similar to those of CD or RPD, several features are worth mentioning. First, the mean SFCT of eyes with both CD and RPD was thinner than the previously reported mean SFCT of eyes with CD and eyes with RPD. It was reported that the mean SFCT was 220.1 – 317.5 µm in eyes with CD, and 174.6 – 186.2 µm in eyes with RPD^2,10,15,17–20^. In the present study, the mean SFCT of eyes with both CD and RPD was 143.8 ± 25.1 µm, which was the thinnest among the three groups. Moreover, it was reported that RPD eyes with neovascular AMD had thinner SFCT (145 ± 48 µm) than RPD eyes with dry AMD (201 ± 88 µm)^21^. Eyes with both CD and RPD appear to have very thin SFCT like RPD eyes with neovascular AMD, although only two from 13 eyes had neovascular AMD. A thin choroid is a characteristic feature of RPD, whereas CD is not related to choroidal thinning^22^. Therefore, the choroid of eyes with both RPD and CD appears to be more severely affected by the degeneration process than that of RPD eyes. Second, Sakurada et al. recently showed that the proportion of CD patients associated with large drusen (> 200 μm) was 34.2%^23^. In a study of Korean patients, although the definition of large drusen was slightly different (at least three times the greatest height or diameter of typical CD), 59.3% of eyes with CD had large drusen^15^. In this study, large drusen (> 200 μm) were accompanied CD and RPD in the same eyes at a much higher rate (92.3%). Third, none of the eyes in the present study had AVLs. In Caucasian patients with CD, 24.2% of eyes reportedly develop vitelliform lesions^2^. However, it has been reported that Asian CD patients rarely have AVLs: only 1.2% of eyes in Korean patients and none in Japanese patients^15,24^. In addition, it has been reported that AVL can be accompanied by RPD^25^. Thus, this small case series also suggests a low prevalence of AVL in Asian patients, even in eyes with both CD and RPD. Fourth, the prevalence of variant SDD in eyes with both CD and RPD (53.8%) was higher than the previously reported prevalence in eyes with RPD (6.6%)^11^. This finding suggests that the microenvironment of eyes with RPD and CD may differ from that of eyes with RPD only. ^9,15^.

We consider that the concurrent presence of CD and RPD may increase the risk of development of late AMD, based on the following findings. The mean SFCT of eyes with both CD and RPD was thinner than the previously reported mean SFCT of eyes with RPD only, and thin choroidal thickness has been reported to be a significant risk factor for developing late AMD in RPD eyes^10^. Large drusen were also found at a much higher rate (92.3%) in eyes with both CD and RPD than in eyes with CD only, and it has been reported that CD eyes associated with large drusen had a high 5-year incidence of late AMD (51.6%)^23^. In addition, there might be an additive effect of individual risk by CD and RPD for the development of late AMD. However, there are some controversies regarding the absence of a diffuse distribution type of RPD, which was a risk factor for the development of late AMD in retrospective studies, and the high accompanying rate of variant SDD, which were suggested as a favorable factor for visual prognosis in a small pilot study^9–11^.

The current study also showed that an eye could have soft drusen, including large drusen, and even variant SDD, in addition to CD and RPD. Previous studies have reported that 79.2% and 6.6% of RPD eyes had soft drusen and variant SDD, respectively^10,11^. In AMD eyes, both soft drusen and RPD were found in 22.1 – 52.1%^26,27^. And, in one study investigating fellow eye of unilateral neovascular AMD, the incidence of soft drusen plus RPD was 13.9%^28^. The coexistence of CD and RPD, with even soft drusen plus variant SDD, indicates that the development of one type of extracellular deposit did not completely interfere with the development of other types of extracellular deposits in AMD eyes. There were overlapping topographic distribution areas, where one type of deposit was intermixed with other types of deposits. However, it remains unclear whether they independently developed or shared some common pathogenetic pathways in their development.

Although little is known about the clinical significance of the coexistence of multiple types of extracellular deposits in AMD eyes, a few previous studies have suggested that the coexistence of soft drusen and RPD might be associated with some differences in the prognosis. Zweifel et al. reported that both soft drusen and RPD were independently correlated with the late AMD^26^. And, one study reported that RPD eyes with other early AMD lesions (soft drusen) in the same eye showed a higher progression rate to late AMD than did RPD eyes with no such lesions (3 years’ follow-up, 18.4% vs. 5%)^10^. Thus, we suppose that individual type of extracellular deposits coexisting in the same eye may be an independent risk factor for late AMD with some additive effect. We hypothesize that AMD eyes with various extracellular deposit profiles have various cellular and extracellular environments, which may have different visual prognosis. Further prospective studies are needed in the future.

The current study had several limitations. The sample size of the cohort was small, and the study was retrospective and cross-sectional. Eyes with a small number of CD and RPD (probably an early form of disease) might not have been included in this cohort, because a minimum number of deposits was required to meet the diagnostic criteria. In addition, eyes with CD in which RPD regressed due to neovascular AMD or outer retinal atrophy after regression of RPD might not have been included in this cohort. In the statistic analysis, there were data skewed in terms of gender, since the majority of the patients included in the study were female. Thus, further studies are needed to investigate the prevalence and clinical features of this disease.

In conclusion, both CD and RPD can develop in the same eye with AMD. Eyes with both CD and RPD had a thinner SFCT and large drusen more frequently than eyes with CD only or eyes with RPD only. Considering the individual risk associated with thin SFCT and the presence of large drusen, eyes with both CD and RPD may have a higher risk of developing late AMD. Therefore, detection of this form of AMD using multimodal imaging may be required for accurate risk assessment. Further studies are needed to investigate the prevalence, clinical features, and prognosis of eyes with both CD and RPD.

## Methods

The clinical records of patients diagnosed with CD or RPD at the retina clinic of Samsung Medical Center, Seoul, Korea, between January 2010 and December 2020, were retrospectively reviewed. We first searched the medical records of patients over 50 years of age diagnosed with CD or RPD. Among them, patients who have undergone multimodal imaging were included in the study if they met the diagnostic criteria for both CD and RPD described below. Cases with poor image quality due to media opacity such as lens opacity were excluded. Exclusion criteria also included traumatic, inflammatory, and hereditary retinal disorders.

Demographic information, including age, sex, ocular and systemic comorbidities, and medical history, were obtained for each patient. All patients underwent a comprehensive ophthalmic examination, including measurement of best-corrected visual acuity (BCVA), refractive error by manifest refraction, slit-lamp biomicroscopy, and fundus examination.

Institutional review board approval was obtained from the Samsung Medical Center institutional review board, and the study was performed in accordance with the principles of the Declaration of Helsinki. Given the retrospective nature of the study and the use of anonymized data, the requirement for informed consent was waived by the institutional review board.

### Imaging analysis

Patients underwent multimodal imaging, including CFP (TRC 50 IX, Topcon, Tokyo, Japan), RF, NIR, FAF, spectral domain-optical coherence tomography (Spectralis HRA + OCT, Heidelberg Engineering, Heidelberg, Germany), and swept-source optical coherence tomography (DRI OCT Triton, Topcon, Tokyo, Japan). In SD-OCT, two B-scans centered on the fovea (horizontal and vertical, 30-degree length, ART 100) and a volume scan (30 × 30-degree square centered on the fovea, 31 horizontal B-scans, ART 25) were obtained. In SS-OCT, two B-scans centered on the fovea (horizontal and vertical, 12 mm, ART 100) and a volume scan (12 × 9 mm square centered on the fovea, 256 horizontal B-scans, ART 4) were obtained. FA, ICGA (Spectralis HRA + OCT or Optos 200Tx, Optos PLC, Dunfermline, UK), and UWF photography (Optos 200Tx) were also performed. Morphological and topographic features of CD and RPD were assessed by two investigators (J.M.Y and D.H.S.), and in case of disagreement, a senior interpreter (D.I.H.) made the final decision.

The presence of soft drusen, large drusen, MNV, GA, drusenoid pigment epithelial detachment (drusenoid PED), and acquired vitelliform lesion (AVL) was determined as follows. Soft drusen are yellowish white, elevated deposits (> 63 µm in size) with slightly blurred boundary in CFP, showing none to minimal hyperfluorescence in later stages of FA. They appear as dome-shaped retinal pigment epithelium (RPE) elevations with homogenous, moderately reflective internal material in OCT. Large drusen were defined as drusen greater than 200 μm, showing RPE elevations in OCT^23^. GA was defined as a sharply demarcated hypopigmented area with visible large choroidal vessels in CFP and hypoautofluorescent in FAF, with a diameter of at least 175 μm. Drusenoid PED was defined as a ≥ ½-disc diameter of confluent soft drusen under the center of the macula^29^. VL was defined as a yellowish subretinal material in color photography, and hyperautofluorescence in FAF, corresponding to dome-shaped hyperreflective material between the ellipsoid zone and the RPE-basal lamina-Bruch’s membrane band on OCT^30,31^.

AMD was classified, according to the Clinical Classification System by the Beckman Initiative for Macular Research Classification Committee. The definition and classification of MNV followed the criteria proposed by the CONAN (Concensus on Neovascular Age-related macular degeneration Nomenclature) study group.

### Diagnosis and distribution of cuticular drusen

The diagnostic criteria for CD used in this study are described in detail elsewhere^15^. In brief, CD were defined as multiple, yellow or pale, small, round lesions observed in CFP, showing a symmetric distribution pattern between bilateral eyes. There had to be at least 50 scattered, uniformly sized, small (25‒75 μm) hyperfluorescent drusen with a typical “stars-in-the-sky” appearance on FA images in each eye^2,5^. The lesion had to be located beneath the RPE, with RPE elevation on OCT images^2,32^.

Fundus topographic distribution patterns of CD were classified as being either the macular or diffuse type, based on CFP and FA results. The macular type was defined as drusen distributed only within the major vascular arcades, whereas the diffuse type was defined as drusen involving the macula, but also extending beyond the vascular arcades^2^.

### Diagnosis and distribution of reticular pseudodrusen

Diagnosis of RPD was based on appropriate findings: (1) multiple yellowish white lesions with a reticular network in CFP, (2) interlacing network in RF imaging, (3) hyporeflectant lesions with mild background hyperreflectance in NIR imaging, (4) hypofluorescent lesions against a background of mild hyperfluorescence in FAF imaging, (5) ≥ 5 hyperreflective subretinal deposits above the RPE on more than one b-scan image in OCT, and (6) hypofluorescent lesions in the mid- or late-phase of ICGA. RPDs were defined as definite if they were identified using at least three imaging methods, including OCT.

The fundus topographic distribution pattern of RPD was classified as localized, intermediate, or diffuse types by the extent of involvement of retinal areas according to criteria described elsewhere^9^.

In addition, variant SDD were defined as deposits with features similar to those of RPD, except for hyperautofluorescence on FAF imaging^11^.

## Supplementary Information

Supplementary Information 1.

Supplementary Information 2.

Supplementary Information 3.

Supplementary Information 4.
