# Supplementary material for: Age-related macular degeneration eyes presenting with cuticular drusen and reticular pseudodrusen
Source: Sci Rep. 2022 Apr 5;12:5681. doi: 10.1038/s41598-022-09608-9 (PMC8983695; doi:10.1038/s41598-022-09608-9)
Supplement: Supplementary file 1 — Supplementary Information 1. [file 41598_2022_9608_MOESM1_ESM.docx]

**Supplementary Table 1.** Demographics and multimodal imaging of patients diagnosed with cuticular drusen or reticular pseudodrusen.

| **Feature** | **Cuticular drusen** | **Reticular pseudodrusen** |
| --- | --- | --- |
| No. of patients | 110 | 463 |
| No. of eyes | 220 | 926 |
| Women | 89 (80.9) | 395 (85.3) |
| Age (years) | 65.8±9.2 | 73.0±8.6 |
| **Multimodal Imaging** | | |
| Color fundus photography | 110 (100) | 463 (100) |
| Optical coherence tomography | 110 (100) | 463 (100) |
| Fundus autofluorescence | 105 (95.5) | 420 (90.7) |
| Near-infrared reflectance | 107 (97.3) | 431 (93.1) |
| Red-free photography | 49 (44.5) | 345 (74.5) |
| Ultrawide-field photography | 94 (85.5) | 399 (86.2) |
| Fluorescein angiography | 110 (100) | 216 (46.7) |
| Indocyanine green angiography | 20 (18.2) | 126 (27.2) |

Data are shown as mean±standard deviation or number (percentage) unless otherwise indicated.

**Supplementary Fig. 1.** Multimodal imagings of a 57-year-old man (Case 4). (**a**) Multiple yellowish deposits are present at the fovea and temporal side of the macula in color fundus photography. Multiple whitish deposits are seen at the superior part of the macula. (**b**) Fundus autofluorescence shows multiple hypofluorescence at the perifoveal and temporal side of the macula. Several hyperfluorescence at the position of large deposits, are also seen. (**c**) Fluorescein angiography (FA) of the venous phase shows numerous hyperfluorescent spots in the macula. (**d**) Indocyanine green angiography of the venous phase also shows hyperfluorescent spots, but less numerous than in FA. (**e**) Horizontal optical coherence tomography (OCT) scan at the fovea shows several sub-retinal pigment epithelial deposits. White arrowheads indicate cuticular drusen, showing hyperfluorescence in FA. Yellow arrowhead indicates soft drusen, showing no fluorescence in FA. (**f**) Many subretinal deposits (blue arrowhead, reticular pseudodrusen) are seen in OCT scan at the superior part of the macula.

**Supplementary Fig. 2.** Multimodal imagings of a 73-year-old woman (Case 6). (**a**) Multiple yellowish and whitish deposits are seen at the central and superior part of the macula in color fundus photography. (**b**) Fluorescein angiography (FA) of the venous phase shows numerous hyperfluorescence of “stars-in-the-sky” appearance. (**c-e**) Optical coherence tomography (OCT) scans show multiple drusen and reticular pseudodrusen (blue arrowhead). Cuticular drusen (white arrowhead) showing hyperfluorescence in FA, soft drusen (yellow arrowhead) showing no fluorescence in FA, and large drusen (yellow arrow, > 200 μm) are seen in OCT scans. (**f, g**) Some of subretinal deposits (green arrowhead) in OCT scan are variant subretinal drusenoid deposits, showing hyporeflective lesions in near-infrared reflectance and hyperfluorescence in fundus autofluorescence.

**Supplementary Fig. 3.** Multimodal imagings of an 80-year-old woman (Case 7). (**a**) Yellowish and whitish deposits are seen in color fundus photography and (**b**) multiple hyperfluorescent spots in fluorescein angiography (FA) of the venous phase. (**c**) Red-free photograph shows numerous deposits of various sizes, and (**d**) fundus autofluorescence shows multiple hypofluorescence, corresponding to cuticular drusen and reticular pseudodrusen, and large soft drusen show mild hyperfluorescence. (**e**) Cuticular drusen (white arrowhead) shows saw-tooth elevation of retinal pigment epithelium in optical coherence tomography (OCT) scan. (**f, g**) Soft drusen (yellow arrowhead), large drusen (yellow arrow, > 200 μm), and reticular pseudodrusen (blue arrowhead) are seen in OCT scan.
