# Supplementary figures and images for: Age-related macular degeneration eyes presenting with cuticular drusen and reticular pseudodrusen
Source: Sci Rep. 2022 Apr 5;12:5681. doi: 10.1038/s41598-022-09608-9 (PMC8983695; doi:10.1038/s41598-022-09608-9)

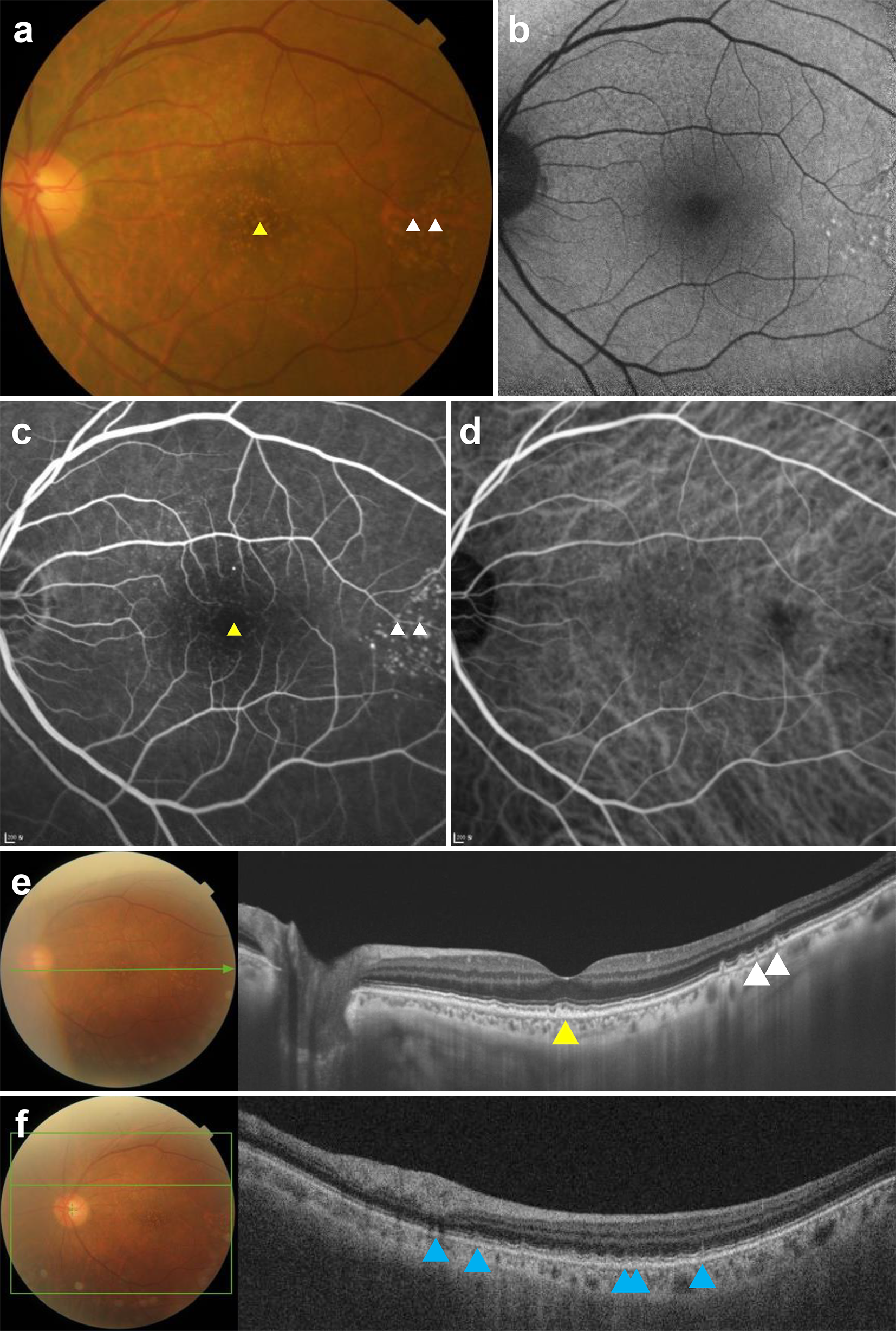

Supplement: Supplementary file 2 — Supplementary Information 2. [file 41598_2022_9608_MOESM2_ESM.tif]

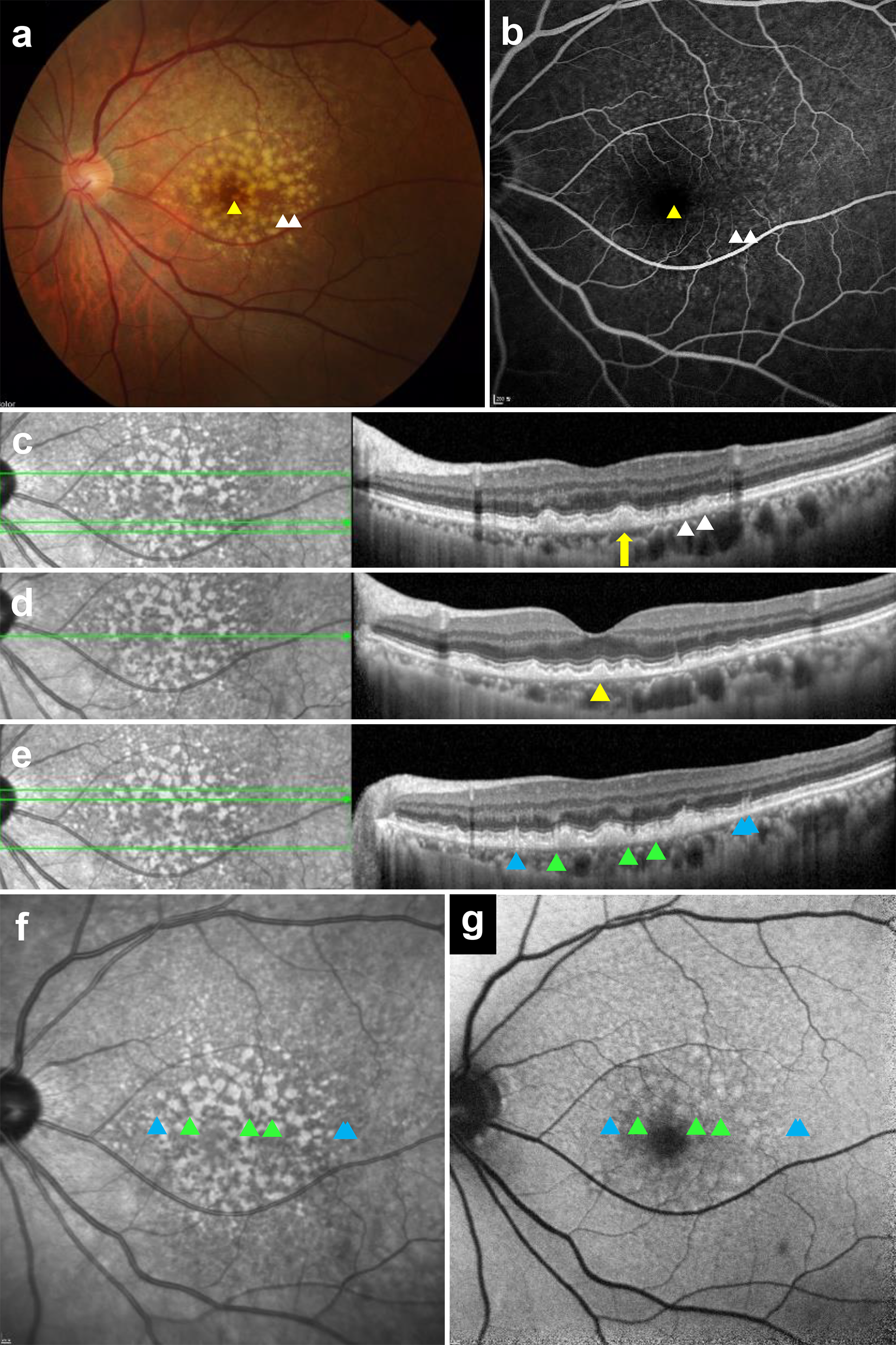

Supplement: Supplementary file 3 — Supplementary Information 3. [file 41598_2022_9608_MOESM3_ESM.tif]

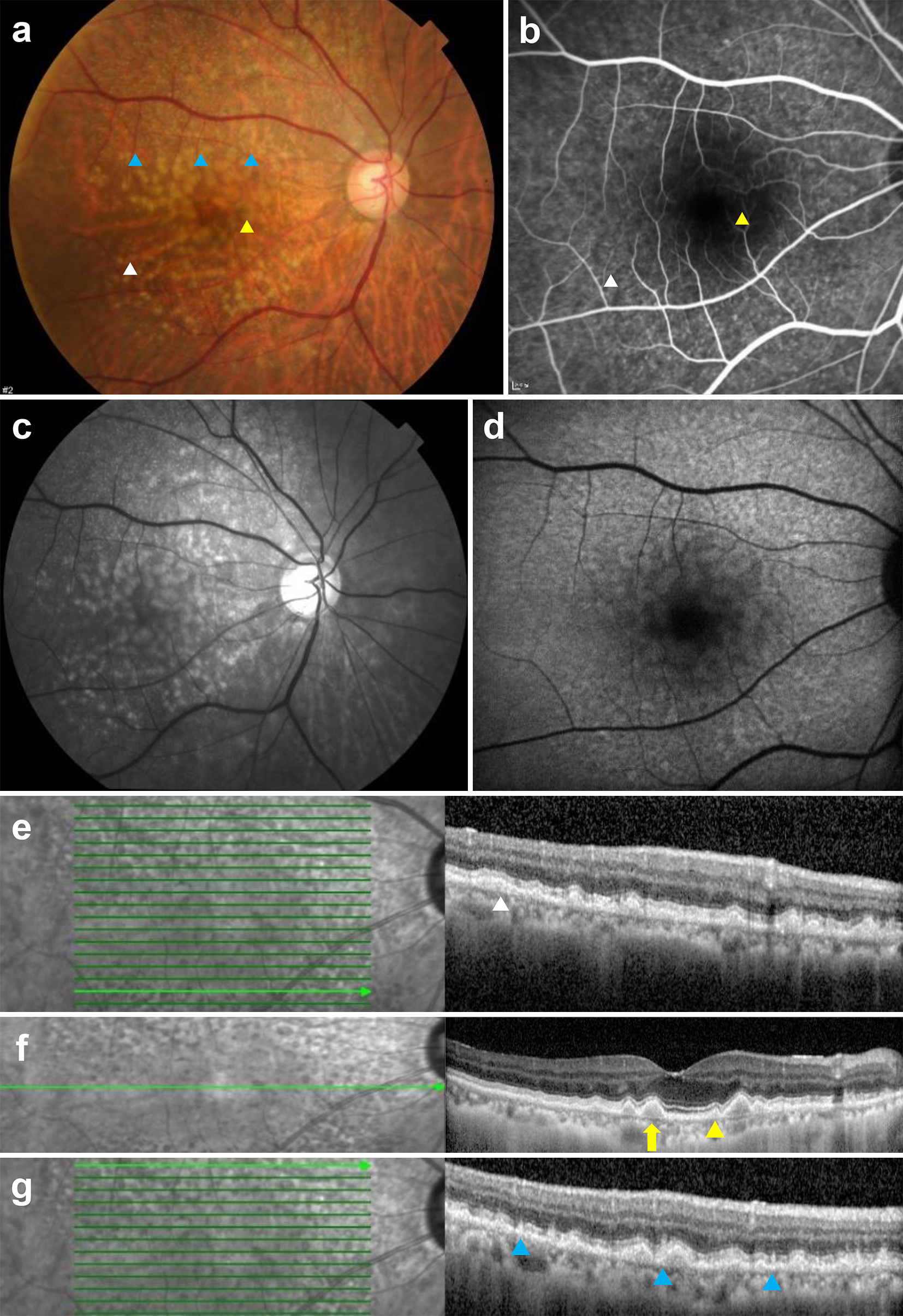

Supplement: Supplementary file 4 — Supplementary Information 4. [file 41598_2022_9608_MOESM4_ESM.tif]
